# COVID-19 Vaccine and Hyperosmolar Hyperglycemic State

**DOI:** 10.7759/cureus.14125

**Published:** 2021-03-26

**Authors:** Mohammed A Abu-Rumaileh, Ahmad M Gharaibeh, Naser Eddin Gharaibeh

**Affiliations:** 1 Endocrinology, University of Jordan School of Medicine, Amman, JOR; 2 Endocrinology, Mercy Hospital Joplin, Joplin, USA; 3 Endocrinology, Kansas City University, Joplin, USA

**Keywords:** covid-19, covid-19 vaccine, hyperosmolar hyperglycemic state, type 2 diabetes, new-onset diabetes

## Abstract

Coronavirus disease 2019 (COVID-19) is a multi-system disease that causes multiple complications. It is linked to the development of new-onset diabetes or unmasking of underlying diabetes. Despite the uncertain exact mechanism, pancreatic angiotensin-converting enzyme 2 (ACE2) receptor, the main enzyme related to COVID-19 pathophysiology has been implied. COVID-19 vaccine was authorized to help control the rapid spread of COVID-19 disease. We report a case of new-onset diabetes type 2 presenting as hyperosmolar hyperglycemic state (HHS) in a patient after receiving COVID-19 vaccine with some literature review of the potential mechanisms by which COVID-19 may cause new-onset diabetes type 2.

## Introduction

Coronavirus disease 2019 (COVID-19) is an infectious disease caused by the novel coronavirus, SARS-CoV-2. This virus was first identified in Wuhan, a city in the Hubei Province of China [1]. Its rapid spread in China has led to an epidemic, followed by a pandemic causing, until the present time, over 118 million cases and over 2.6 million deaths [2]. COVID-19 is a multi-system disease thought to be linked to development of new-onset diabetes [3]. The pressing need for a vaccine to help control this pandemic accelerated an Emergency Use Authorization (EUA) for Pfizer-BioNTech COVID-19 Vaccine in December 2020 [4]. We report a case of hyperosmolar hyperglycemic state (HHS) occurring post administration of Pfizer-BioNTech COVID19 Vaccine.

## Case presentation

A 58-year-old African male with a history of hypertension presented to the Emergency Room on 01/14/2021 complaining of progressive altered mental status of three days duration. The patient reported receiving his first Pfizer-BioNTech COVID-19 vaccine dose on 12/18/2020, after which he noticed increased nocturia to four times nightly, where he used to have nocturia only twice at night for the last few months, before the administration of the vaccine. 

On 01/08/2021, the patient received his second Pfizer-BioNTech COVID-19 vaccine dose, two days afterwards, his nocturia significantly increased to around 12 times nightly. In addition, he noticed polyuria and polydipsia. He felt severely dehydrated and started drinking large amounts of fluids including 12 cans of regular soda and continued to have worsening mental status over the next three days. He lost 20 pounds in one-week period.

The patient denied any symptoms to suggest infection, such as hesitancy, urgency, or abdominal pain. He also denied cough, shortness of breath, fever, chills, or night sweats. 

Upon presentation to the Emergency Room on 01/14/2021, the patient was drowsy, severely dehydrated, and disoriented. His blood pressure was 143/76 mmHg with heart rate of 65 beats per minute, he was ill-appearing with mild tachypnea but no other significant findings.

His initial laboratory results showed serum glucose level of 1253 mg/dL, bicarbonate level of 24 mmol/L with β-hydroxybutyrate level of 8.5 mmol/L. Venous blood gas with other relevant laboratory results are shown in Tables 1, 2.

**Table 1:** Blood chemistry studies and CBC. CBC: complete blood count; WBC: white blood cell; AST: aspartate transaminase; ALT: alanine aminotransferase.

Blood chemistry studies	Admission lab 01/14/2021	01/19/2021	One-week post-discharge
Sodium (136-145 mmol/L)	144	140	133
Potassium (3.5-5.1 mmol/L)	5.4	3.2	4.3
Chloride (98-107 mmol/L)	95	105	104
Bicarbonate (22-29 mmol/L)	24	25	20
Calcium (8.8-10.2 mmol/L)	10.2	8.5	8.9
Blood urea nitrogen (8-23 mmol/L)	60	19	13
Creatinine (0.67-1.17 mg/dL)	1.95	1.13	1
Glomerular filtration rate (mL/min/1.73 sq meter)	36	>60	>60
Glucose (74-99 mg/dL)	1253	117	184
AST (0-40 U/L)	33	54	35
ALT (0-41 U/L)	62	55	49
Anion gap (4-13 mmol/L)	25	10	9
CBC			
Hemoglobin (13.5-18 g/dL)	15.8	13.6	12.4
Platelets (1.5-4.5 x 10^5^/L)	127	54	183
WBC (4.5-11.0 x 10^9^/L)	11.9	7.8	7.1
Neutrophils %	80	49	56
Lymphocytes %	16	43	35

**Table 2:** Other labs. TSH: thyroid-stimulating hormone; LDL: low-density lipoprotein; HDL: high-density lipoprotein.

Venous blood gases	During hospitalization
Specimen source	Venous
pH blood (7.38-7.46)	7.35
pCO2 (32-46 mmHg)	47
Bicarbonate (21-29 mmol/L)	26
pO2 (25-40 mmHg)	25
O2 Sat	42
TCO2 (22-31 mmol/L)	27
Base excess (-2 - 2 mmol/L)	0
Miscellaneous	
HBA1C (<=5.6%)	13
ß-Hydroxybutyrate (<0.3 mmol/L)	8.5
Glutamic acid decarboxylase anti-body (<=0.02 nmol/L)	0
Islet cell AB (<=0.02 nmol/L)	0
C-peptide (1.10-4.40 ng/ml)	1.1
Osmolality (275-300 mOsm/kg)	371
TSH (0.27-4.2 ulU/ml)	0.5
Vitamin D	30
Troponin T, 5th Gen	14
Lipid profile	
Cholesterol (<200 mg/dL)	239
HDL (40-59 mg/dL)	54
LDL (<100 mg/dL)	128
Non-HDL Cholesterol (<130 mg/dL)	185
Triglyceride (>150 mg/dL)	285

The patient was admitted to the Intensive Care Unit, started on intravenous fluids with insulin drip. Next day, he was transitioned to multiple daily injections regimen and transferred to the floor for further management. His insulin regimen was up titrated and good glycemic control was achieved on insulin glargine 50 units once daily, in addition to 10 units of pre-meal insulin with correction scale.

PCR test was negative for COVID-19, Respiratory Syncytial Virus, and Influenza A, B. Chest x-ray was clear, EKG was normal, urine culture showed no growth.

The patient has family history of diabetes type 2 in both of his parents, he also has personal history of skin tags in his neck that started a few years ago, he reported that his diet was moderate in carbohydrates. Upon review of his chart, his fasting glucose has ranged from 74-120 in the last three years (Table 3). However, no baseline hemoglobin A1C was available. Four weeks after discharge, the dose of insulin was slowly tapered down then discontinued. The patient is currently only on metformin with excellent glycemic control, his fasting serum blood glucose level is 73 mg/dL, with C-peptide level of 3.65 ng/ml.

**Table 3:** Previous serum blood glucose readings.

Date	08/24/2020	06/01/2020	02/11/2020	02/06/2019	11/14/2017	05/15/2017
Glucose (74-99 mg/dL)	80	74	120	88	106	118

## Discussion

Diabetes mellitus type 2 (DM-II) is a chronic disorder characterized by high blood glucose levels during a prolonged period of time. The natural history of DM-II starts with normal glucose tolerance with subsequent insulin resistance, hyperinsulinemia, then impaired glucose tolerance and finally DM-II [5].

HHS is a serious complication of DM-II, that can be fatal if untreated. Predisposing conditions include infection or inadequate insulin therapy. HHS results in extremely elevated glucose levels from inefficient insulin and mild elevation of counter-regulatory hormones (glucagon, catecholamines, cortisol, and growth hormone) that cause increased hepatic glucose production. Patients with HHS present with extreme thirst, frequent urination, confusion, weakness, and sometimes coma. HHS is diagnosed with blood glucose level above 600 mg/dL, plasma osmolality >320, in the absence of significant ketoacidosis [6]. 

Hyperglycemia (typically with glucose level around 200 mg/dl) causes glycosuria [7] which causes a net loss of water and dehydration. Severe dehydration and high serum osmolality are the main causes of altered mental status in patients at presentation [8]. HHS is characterized by inadequate insulin levels which will slightly increase lipolysis and ketogenesis, mechanisms through which blood ketone levels may mildly increase in patients with this condition [6]. 

A meta‐analysis of eight different studies of nearly 3,700 patients revealed an average of 14.4% for new-onset diabetes on admission in patients hospitalized with COVID-19 [9]. Similar results were found in a study of 453 patients admitted with COVID-19. Upon admission, 20.7% met the diagnostic criteria of new-onset diabetes. Patients with new-onset diabetes on admission for COVID-19 were found to have a significant increase in mortality compared with patients with prediabetes or preexisting diabetes [10].

SARS-CoV-2 virus pathogenesis involves viral S protein utilization of cellular angiotensin-converting enzyme 2 (ACE2) receptor [11]. As ACE2 is expressed in key organs that regulate glucose metabolism such as pancreas, adipose tissue, and kidneys, it is thought that SARS-CoV-2 could impair glucose homeostasis [3]. In an experimental mice study, removal of ACE2 resulted in impairment of glucose homeostasis predisposing them to DM-II [12]. Studies documented that SARS-CoV-1 utilizes the ACE2 receptors and damages pancreatic β-cells causing acute diabetes [13]. 

Multiple studies confirmed the expression of ACE2 receptors in the β-cells of pancreas making them prone to SARS-CoV-2 infection [11]. Entry of SARS-CoV-2 into pancreatic β-cells was detected in vitro, indicating possible destruction of pancreatic cells upon entry of the virus [14].

Renin-angiotensin system (RAS) is an enzyme system cascade that produces angiotensin II which promotes vasoconstriction, oxidative stress, inflammation, in addition to salt and water reabsorption [15]. ACE2 metabolizes angiotensin II, thereby down-regulating the activity of RAS pathway [16].

RAS components were found in several tissues including pancreas, liver, and muscles. RAS hyperactivity decreases perfusion of the pancreatic tissue and reduces insulin production, and increases oxidative stress resulting in fibrosis. Also, it decreases insulin sensitivity on target tissues [17]. SARS-CoV viral entry to ACE2 expressing cells causes down-regulation of ACE2 which increases RAS pathway activity [18], possibly causing worsened insulin resistance and decreased insulin release [17]. Interestingly, ACE2 receptors are expressed on the surface of macrophages [19]. Infection of islet macrophages may impair pancreatic β-cells function [20].

## Conclusions

While Pfizer-BioNTech COVID-19 vaccine appears to be relatively safe, the vaccine might trigger an immune response that may have unmasked the patient’s underlying pre-diabetes. As of the time of writing this report, no cases of new-onset diabetes after COVID-19 vaccine were found in the literature. Based on this report, regulatory bodies should consider close monitoring of COVID-19 vaccines effect on metabolic profile including the risk of new-onset DM-II. In addition, prompt screening for DM-II in susceptible patients following administration of COVID-19 vaccine should be considered by treating providers.
